# Supplementary material for: Let’s talk about pain catastrophizing measures: an item content analysis
Source: PeerJ. 2020 Mar 4;8:e8643. doi: 10.7717/peerj.8643 (PMC7060750; doi:10.7717/peerj.8643)
Supplement: Table S1 — ∗p < 0.05, indicating that the score of an item for that particular construct is significantly different from the score for the construct ’Pain catastrophizing.’ [file peerj-08-8643-s001.docx]

| Item | Measure | Pain catastrophizing $\hat{\boldsymbol{\mu}}$[CI] | Pain-related disability $\hat{\boldsymbol{\mu}}$ [CI] | Pain-related distress $\hat{\boldsymbol{\mu}}$ [CI] | Pain severity $\hat{\boldsymbol{\mu}}$[CI] | Pain vigilance $\hat{\boldsymbol{\mu}}$[CI] | Worry about pain $\hat{\boldsymbol{\mu}}$ [CI] |
| --- | --- | --- | --- | --- | --- | --- | --- |
| *When I become aware of my pain, this thought comes through my head: "I can't have a tumour, can I?"* | AEQ | 5.43  [3.01 to 7.84] | -4.78*  [-7.07 to -2.50] | 4.46  [2.17 to 6.75] | -4.55*  [-6.93 to -2.17] | -3.55*  [-6.04 to -1.06] | 6.49  [4.57 to 8.41] |
| *When I become aware of my pain, this thought comes through my head: "I wonder if I have the same serious illness as…"* | AEQ | 4.48  [1.61 to 7.35] | -7.92*  [-9.09 to -6.77] | 3.08  [0.14 to 6.03] | -5.50*  [-8.01 to -2.96] | -3.09*  [-6.06 to -0.12] | 4.19  [1.30 to 7.07] |
| *When I become aware of my pain, this thought comes through my head: "it isn't a serious illness, is it?"* | AEQ | 3.70  [2.12 to 5.27] | -6.16*  [-7.24 to -5.07] | 3.50  [1.95 to 5.04] | -4.02*  [-5.41 to -2.61] | -1.81*  [-3.36 to -0.23] | 6.21*  [4.97 to 7.44] |
| *I feel like I just want to get up and run away* | CCSI | -0.97  [-3.43 to 1.47] | -4.46*  [-6.49 to -2.45] | 3.03*  [0.50 to 5.53] | -3.26  [-5.59 to -0.91] | -4.94*  [-6.97 to -2.89] | 0.59  [-2.12 to 3.30] |
| *I begin thinking of all the possible bad things that could go wrong in association with the pain* | CCSI | 3.93  [0.89 to 6.95] | -6.57*  [-8.58 to -4.58] | 4.40  [1.52 to 7.26] | -5.71*  [-8.16 to -3.24] | -4.06*  [-6.86 to -1.27] | 5.92  [3.36 to 8.49] |
| *I find myself worrying about possibly dying* | CCSI | 4.14  [1.53 to 6.77] | -4.46*  [-6.65 to -2.26] | 3.02  [0.22 to 5.79] | -2.64*  [-5.17 to -0.12] | -1.30*  [-4.03 to 1.44] | 5.55  [3.37 to 7.74] |
| *I find myself expecting the worst* | CCSI | 5.09  [3.71 to 6.46] | -6.21*  [-7.25 to -5.17] | 2.87*  [1.30 to 4.41] | -4.43*  [-5.75 to -3.09] | -1.13*  [-2.74 to 0.51] | 4.98  [3.59 to 6.35] |
| *I tend to think that my pain is pretty awful* | CCSI | 4.76  [2.06 to 7.42] | -7.99*  [-9.35 to -6.43] | 2.75  [-0.13 to 5.61] | 4.10  [1.21 to 6.99] | -5.58*  [-8.02 to -3.14] | 0.45*  [-2.76 to 3.66] |
| *I can't help but concentrate on how bad the pain actually feels* | CCSI | -0.92  [-4.05 to 2.21] | -7.00*  [-8.52 to -5.48] | 4.88*  [2.20 to 7.56] | 4.61*  [1.74 to 7.49] | -0.20  [-3.36 to 2.92] | 1.24  [-1.95 to 4.44] |
| *I find it virtually impossible to keep my mind off of my pain and how bad it hurts* | CCSI | 0.49  [-2.51 to 3.49] | 0.32  [-2.62 to 3.22] | 7.73*  [5.96 to 9.49] | 7.12*  [5.07 to 9.17] | 0.60  [-2.39 to 3.59] | 4.76*  [2.19 to 7.33] |
| *I begin to worry that something might be seriously wrong with me* | CCSI | 2.08  [-0.57 to 4.72] | -5.87*  [-7.57 to -4.18] | 2.58  [-0.05 to 5.17] | -4.49*  [-6.66 to -2.31] | -2.85*  [-5.26 to -0.45] | 5.93*  [3.79 to 8.08] |
| *When I feel pain, I feel my life isn't worth living* | CSQ | 5.90  [4.64 to 7.15] | -2.21*  [-3.73 to -0.67] | 8.28*  [7.58 to 8.99] | 0.54*  [-1.11 to 2.20] | -4.50*  [-5.80 to -3.17] | 3.89*  [2.35 to 5.43] |
| *When I'm in pain, I worry all the time about whether the pain will end* | PCS/CSQ | -0.29  [-3.01 to 2.44] | -4.61*  [-6.82 to -2.40] | 6.02*  [4.13 to 7.91] | -1.11  [-3.71 to 1.49] | -0.47  [-3.18 to 2.24] | 8.44*  [7.31 to 9.57] |
| *When I'm in pain, I feel I can't go on* | PCS/CSQ | 2.31  [-0.84 to 5.49] | 0.25  [-3.03 to 3.50] | 7.20*  [5.17 to 9.20] | 1.07  [-1.90 to 4.04] | -6.55*  [-8.51 to -4.59] | 2.24  [-0.78 to 5.26] |
| *When I'm in pain, it's terrible and I think it's never going to get any better* | PCS/CSQ | 7.41  [5.36 to 9.44] | -3.56*  [-6.39 to -0.75] | 7.21  [5.41 to 9.00] | 4.70  [2.14 to 7.26] | -1.62*  [-4.55 to 1.31] | 6.21  [3.76 to 8.65] |
| *When I'm in pain, it's awful and I feel that it overwhelms me* | PCS/CSQ | 3.17  [0.61 to 5.71] | 0.65  [-1.85 to 3.12] | 7.02*  [5.45 to 5.59] | 5.26  [3.17 to 7.35] | -3.56*  [-5.86 to -1.27] | 2.53  [-0.07 to 5.12] |
| *When I'm in pain, I feel I can't stand it anymore* | PCS/CSQ | 5.49  [3.37 to 7.62] | -0.49*  [-3.35 to 2.34] | 6.82  [5.15 to 8.46] | 5.32  [2.99 to 7.64] | -5.00*  [-7.11 to -2.88] | 1.88*  [-0.89 to 4.66] |
| *When I'm in pain, I become afraid that the pain will get worse* | PCS | 2.20  [-1.04 to 5.43] | -6.99*  [-8.83 to -5.15] | 4.26  [1.56 to 6.95] | 1.55  [-1.50 to 4.58] | -0.17  [-3.32 to 2.96] | 8.27*  [6.70 to 9.84] |
| *When I'm in pain, I keep thinking of other painful events* | PCS | -0.88  [-4.05 to 2.30] | -6.40*  [-8.73 to -4.07] | 6.61*  [4.36 to 8.84] | -6.78*  [-8.85 to -4.71] | -5.82*  [-8.10 to -3.54] | 4.13*  [1.23 to 7.04] |
| *When I'm in pain, I anxiously want the pain to go away* | PCS | -6.54  [-8.36 to -4.69] | -6.92  [-8.85 to -4.99] | 5.61*  [3.03 to 8.18] | -3.74*  [-6.72 to -0.77] | -1.68*  [-4.79 to 1.41] | 6.27*  [3.84 to 8.71] |
| *When I'm in pain, I can't seem to keep it out of my mind* | PCS | -0.97  [-3.70 to 1.77] | -0.85  [-3.51 to 1.79] | 6.43*  [4.43 to 8.42] | -0.96  [-3.57 to 1.66] | -1.59  [-4.21 to 1.03] | 5.64*  [3.33 to 7.96] |
| *When I'm in pain, I keep thinking about how much it hurts* | PCS | 0.46  [-2.53 to 3.46] | -3.22  [-5.94 to -0.54] | 6.37*  [4.22 to 8.51] | 6.90*  [5.05 to 8.75] | 2.76  [-0.05 to 5.57] | 7.12*  [5.29 to 8.95] |
| *When I'm in pain, I keep thinking about how badly I want the pain to stop* | PCS | -2.54  [-5.43 to 0.37] | -3.41  [-6.09 to -0.74] | 8.57*  [7.31 to 9.83] | 4.61*  [2.02 to 7.21] | 0.12  [-2.87 to 3.11] | 5.35*  [2.84 to 7.84] |
| *When I'm in pain, there's nothing I can do to reduce the intensity of the pain* | PCS | 1.17  [-1.58 to 3.92] | -2.43  [-5.02 to 0.12] | 3.84  [1.27 to 6.38] | 5.79*  [3.57 to 8.01] | -5.12*  [-7.24 to -3.00] | 1.94  [-0.88 to 4.76] |
| *When I'm in pain, I wonder whether something serious may happen* | PCS | 3.46  [1.90 to 5.01] | -6.00*  [-7.09 to -4.90] | 5.71*  [4.47 to 6.93] | -4.77*  [-6.08 to -3.44] | -1.93*  [-3.54 to -0.29] | 7.03*  [5.89 to 8.16] |
| *I often think back to the days when I had no pain* | PCL | -3.63  [-6.18 to -1.05] | -4.76  [-7.20 to -2.33] | -0.16*  [-3.09 to 2.76] | -2.05  [-4.85 to 0.74] | -4.77  [-7.13 to -2.40] | -0.40*  [-3.42 to 2.61] |
| *I focus on the pain all the time* | PCL | -3.39  [-6.21 to -0.53] | -5.99  [-8.30 to -3.70] | 0.99*  [-2.32 to 4.30] | -2.99  [-6.03 to 0.05] | 3.85*  [0.76 to 6.93] | 5.30*  [2.65 to 7.96] |
| *I consider myself an unlucky person* | PCL | -2.01  [-4.99 to 0.95] | -6.20*  [-8.26 to 4.17] | -3.45  [-6.35 to -0.59] | -5.91*  [-8.12 to -3.69] | -6.40*  [-8.33 to -4.48] | -3.68  [-6.35 to -1.02] |
| *I think that fate has caught up with me* | PCL | -2.60  [-5.58 to 0.38] | -6.93*  [-8.55 to -5.31] | -0.35  [-3.31 to 2.59] | -7.61*  [-8.91 to -6.30] | -6.38*  [-8.40 to -4.37] | 0.78  [-2.15 to 3.71] |
| *The word pain frightens me* | PCL | -1.05  [-3.81 to 1.72] | -5.87*  [-7.81 to -3.94] | 2.49*  [-0.28 to 5.22] | -5.52*  [-7.56 to -3.47] | -3.44  [-5.96 to -0.92] | 5.79*  [3.71 to 7.86] |
| *I often think: 'Why is this happening to me?'* | PCL | -2.26  [-5.26 to 0.75] | -5.16  [-7.67 to -2.66] | 4.09*  [1.27 to 6.93] | -4.71  [-7.43 to -1.99] | -6.09*  [-8.41 to -3.79] | 3.38*  [0.50 to 6.25] |
| *I think that, because of the pain, I have changed my view of life* | PCL | -3.53  [-6.48 to -0.59] | -2.71  [-5.54 to 0.10] | 0.79*  [-2.46 to 4.03] | -5.19  [-7.86 to -2.51] | -3.95  [-6.56 to -1.34] | -2.50  [-5.60 to 0.60] |
| *I feel powerless against the pain* | PCL | -0.03  [-2.95 to 2.89] | -2.28  [-5.03 to 0.46] | 6.54*  [4.55 to 8.51] | 0.42  [-2.40 to 3.25] | -3.35  [-6.01 to -0.70] | 5.93*  [3.55 to 8.30] |
| *I think I am always very tense* | PCL | -5.51  [-7.48 to -3.54] | -6.04  [-7.79 to -4.30] | -0.17*  [-2.96 to 2.60] | -5.93  [-7.83 to -4.02] | -3.37*  [-5.95 to -0.79] | 1.31*  [-1.33 to 3.94] |
| *I make sure I protect myself from extra pain* | PCL | -5.55  [-6.64 to -4.46] | -5.40  [-6.58 to -4.21] | -2.46*  [3.98 to -0.94] | -2.10*  [-3.69 to -0.52] | 6.91*  [-5.81 to 8.04] | 1.18*  [-0.41 to 2.77] |
| *I have become less able mentally* | PCL | -3.49  [-5.73 to -1.27] | 2.50*  [-0.22 to 5.18] | 1.82*  [-0.69 to 4.31] | -3.43  [-5.79 to -1.07] | -5.20  [-7.21 to -3.18] | -1.67  [-4.25 to 0.92] |
| *I consider myself to be a hopeless case* | PCL | 1.97  [-1.27 to 5.22] | -4.33*  [-7.11 to -1.55] | 2.27  [-0.97 to 5.50] | -5.23*  [-7.74 to -2.73] | -7.02*  [-8.89 to 5.16] | -2.37*  [-5.55 to 0.82] |
| *When the pain is very strong I get furious* | PCL | 0.80  [-1.86 to 3.49] | -4.34*  [-6.66 to -2.03] | 7.05*  [-5.35 to 8.74] | 5.26*  [2.95 to 7.57] | -4.53*  [-6.73 to -2.32] | 0.09  [-2.68 to 2.86] |
| *I sometimes wonder how other people cope with severe pain* | PCL | -4.21  [-6.60 to -1.80] | -4.44  [-7.02 to -1.88] | -0.80*  [-3.76 to 2.15] | 0.37*  [-2.61 to 3.36] | -2.77  [-5.43 to -0.12] | 4.13*  [1.59 to 6.66] |
| *I cannot live without pain-killers* | PCL | 0.93  [-2.29 to 4.17] | -0.55  [-3.65 to 2.51] | 1.44  [-1.75 to 4.62] | 4.12  [1.27 to 6.98] | -0.71  [-3.71 to 2.27] | 0.39  [-2.62 to 3.39] |
| *I am disappointed in myself for giving in to the pain* | PCL | -4.88  [-7.33 to -2.41] | -4.64  [-7.16 to -2.15] | 4.57*  [1.79 to 7.35] | -4.57  [-7.33 to -1.78] | -5.88  [-8.25 to -3.53] | 0.59*  [-2.47 to 3.66] |
| *I cannot stand this pain any longer* | PRSS | 3.48  [0.78 to 6.19] | -2.03*  [-4.82 to 0.74] | 7.11*  [-5.07 to 9.17] | 7.51*  [5.84 to 9.18] | -3.03*  [-5.66 to -0.41] | 4.35  [1.70 to 6.99] |
| *No matter what I do, my pain doesn't change anyway* | PRSS | 0.85  [-1.93 to 3.63] | -3.14*  [-5.64 to -0.67] | 0.56  [-2.21 to 3.28] | 1.18  [-1.58 to 3.93] | -1.62  [-4.35 to 1.10] | -0.55  [-3.35 to 2.25] |
| *I need to take some pain medication* | PRSS | -7.06 [-8.68 to -5.43] | -3.57*  [-6.49 to -0.68] | -1.71*  [-4.73 to 1.30] | 2.97*  [-0.02 to 5.96] | -0.20*  [-3.43 to 3.01] | -3.09  [-5.96 to -0.20] |
| *This will never end* | PRSS | 6.58  [4.51 to 8.64] | -4.61*  [-7.23 to -2.00] | 5.74  [3.23 to 8.24] | -0.75*  [-3.88 to 2.36] | -4.58*  [-7.28 to -1.90] | 4.75  [1.91 to 7.59] |
| *I am a hopeless case* | PRSS | 1.79  [-0.90 to 4.49] | -4.66*  [-6.85 to -2.52] | 2.03  [-0.69 to 4.72] | -4.61*  [-6.72 to -2.50] | -5.18*  [-7.05 to -3.29] | 1.62  [-1.05 to 4.30] |
| *When will it get worse again?* | PRSS | -2.26  [-5.38 to 0.89] | -6.99*  [-8.91 to -5.09] | 1.58*  [-1.68 to 4.81] | -0.20  [-3.33 to 2.93] | 5.58*  [3.10 to 8.03] | 7.13*  [5.16 to 9.11] |
| *This pain is killing me* | PRSS | 5.04  [3.64 to 6.44] | -0.72*  [-2.35 to 0.92] | 5.76  [4.46 to 7.05] | 6.73  [5.56 to 7.90] | -4.19*  [-5.59 to -2.77] | 1.89*  [0.27 to 3.51] |
| *I can't go on anymore* | PRSS | 5.92  [3.60 to 8.24] | 2.58  [-0.33 to 5.48] | 8.05*  [6.33 to 9.76] | 3.54  [0.66 to 6.41] | -5.48*  [-7.45 to -3.52] | 3.63  [0.88 to 6.38] |
| *This pain drives me crazy* | PRSS | 1.84  [-1.04 to 4.73] | -0.15  [-3.08 to 2.73] | 7.12*  [5.11 to 9.12] | 7.22*  [5.35 to 9.09] | -2.55*  [-5.43 to 0.33] | 2.61  [-0.33 to 5.54] |
