# Supplementary material for: Let’s talk about pain catastrophizing measures: an item content analysis
Source: PeerJ. 2020 Mar 4;8:e8643. doi: 10.7717/peerj.8643 (PMC7060750; doi:10.7717/peerj.8643)
Supplement: File S1 [file peerj-08-8643-s002.docx]

*Worrying about pain*. The items of the PCI, designed to measure ‘worrying about pain’, scored significantly higher on pain-related worrying about pain ($\hat{\mu}$ *=* 3.05, 95% CI [1.18 to 4.79]) than on pain catastrophizing ($\hat{\mu}$ *=* -2.35, 95% CI [-4.20 to -0.62]) (Δ = 5.40, 95% CI [4.36 to 6.44]), pain-related disability ($\hat{\mu}$ = -3.29, 95% CI [-5.16 to -1.56])(Δ = 6.34, 95% CI [5.32 to 7.37]), pain severity ($\hat{\mu}$ = -2.49, 95% CI [-4.36 to -0.75])(Δ = 5.54, 95% CI [4.51 to 6.58]) and pain vigilance ($\hat{\mu}$ = -1.97, 95% CI [-3.84 to -0.24]) (Δ = 5.02, 95% CI [4.00 to 6.05]). The difference with pain-related distress did however not reach significance ($\hat{\mu}$ = 2.09, 95% CI [0.22 to 3.82]) (Δ = 0.96, 95% CI [0.08 to 1.99]).

*Pain vigilance*. The items of the PVAQ, designed to measure ‘pain vigilance’, scored significantly higher on pain vigilance ($\hat{\mu}$ *=* 4.56, 95% CI [2.67 to 6.57]) than on pain catastrophizing ($\hat{\mu}$ = -3.10, 95% CI [-5.00 to -1.09]) (Δ = 7.66, 95% CI [6.64 to 8.69]), pain-related disability ($\hat{\mu}$ = -4.49, 95% CI [-6.38 to -2.48]) (Δ = 9.05, 95% CI [8.01 to 10.09]), pain-related distress ($\hat{\mu}$ = -0.03, 95% CI [-1.93 to 2.01]) (Δ = 4.58, 95% CI [3.55 to 5.62]), pain severity ($\hat{\mu}$ = 0.78, 95% CI [-1.11 to 2.80]) (Δ = 3.77, 95% CI [2.74 to 4.80]) and worrying about pain ($\hat{\mu}$ = 0.72, 95% CI [-1.18 to 2.74]) (Δ = 3.84, 95% CI [2.80 to 4.88]).

*Pain-related distress*. The items of the affective distress subscale of the MPI, scored significantly higher on pain-related distress ($\hat{\mu}$ *=* 2.22, 95% CI [-2.05 to 8.25]) than on pain catastrophizing ($\hat{\mu}$ = -4.78, 95% CI [-8.98 to 1.24]) (Δ = 7.00, 95% CI [4.74 to 9.25]), pain-related disability ($\hat{\mu}$ = -4.05, 95% CI [-8.33 to 1.99]) (Δ = 6.26, 95% CI [4.01 to 8.51]), pain severity ($\hat{\mu}$ = -4.89, 95% CI [-9.09 to 1.12]) (Δ = 7.11, 95% CI [4.84 to 9.35]) and pain vigilance ($\hat{\mu}$ = -2.35, 95% CI [-6.65 to 3.68]) (Δ = 4.57, 95% CI [2.31 to 6.83]). The difference with pain-related worrying was not significant ($\hat{\mu}$ = 1.07, 95% CI [-3.20 to 7.11]) (Δ = 1.14, 95% CI [-1.11 to 3.39]).

*Pain-related disability*. The items of the disability subscale of the MPI, scored significantly higher on pain-related disability ($\hat{\mu}$ *=* 4.24, 95% CI [2.94 to 5.53]) than on pain catastrophizing ($\hat{\mu}$ = -5.35, 95% CI [-6.66 to -4.06]) (Δ = 9.60, 95% CI [8.65 to 10.54]), pain-related distress ($\hat{\mu}$ = 0.02, 95% CI [-1.28 to 1.31]) (Δ = 4.23, 95% CI [3.28 to 5.18]), pain severity ($\hat{\mu}$ = -1.40, 95% CI [-2.68 to -0.11]) (Δ = 5.64, 95% CI [4.69 to 6.59]), pain vigilance ($\hat{\mu}$ = -3.78, 95% CI [-5.08 to -2.48]) (Δ = 8.02, 95% CI [7.07 to 8.97]) and worrying about pain ($\hat{\mu}$ = -2.34, 95% CI [-3.64 to -1.04]) (Δ = 6.59, 95% CI [5.63 to 7.53]).

*Pain severity*. The items of the pain severity subscale of the MPI scored significantly higher on pain severity ($\hat{\mu}$ *=* 6.36, 95% CI [2.51 to 9.47]) than on pain catastrophizing ($\hat{\mu}$ = -4.52, 95% CI [-8.40 to -1.33]) (Δ = 10.88, 95% CI [9.44 to 12.32), pain-related disability ($\hat{\mu}$ = -4.22, 95% CI [-8.13 to -1.02]) (Δ = 10.58, 95% CI [9.15 to 12.01), pain-related distress ($\hat{\mu}$ = -0.73, 95% CI [-4.61 to 2.48]) (Δ = 7.09, 95% CI [5.66 to 8.52), pain vigilance ($\hat{\mu}$ = -0.70, 95% CI [-4.59 to 2.50]) (Δ = 7.06, 95% CI [5.63 to 8.49) and worrying about pain ($\hat{\mu}$ = -2.96, 95% CI [-6.84 to 0.26]) (Δ = 9.32, 95% CI [7.89 to 10.75]).
