# Supplementary material for: Let’s talk about pain catastrophizing measures: an item content analysis
Source: PeerJ. 2020 Mar 4;8:e8643. doi: 10.7717/peerj.8643 (PMC7060750; doi:10.7717/peerj.8643)

1. **Comparability between the three groups.**

To check whether participants from the three groups (each receiving one of the three item sets) were comparable with respect to their scores, we analyzed the responses to the shared items with linear mixed effect models as implemented in the R package lmerTest (version 3.1.0; Kuznetsova, Brockhoff, & Christensen, 2017). Satterthwaite’s approximation was used to obtain the degrees of freedom (SAS Technical Report R-101, 1978). Model assumptions of  independence, normality and homogeneity of variance were checked. Significance was evaluated at the 5% significance level.

A model was fitted with *construct*, *item set group* and their interactions as fixed effects and a random *subject*, *item* and *measure* effect. We chose to include *measure* as random effect, because the measures included in this study are only a part of the total population of questionnaires. There was a significant main effect of *construct* (F(5,5521.8) = 65.38, *p* < 0.001), which was not of interest here. Both the main effect of *item set* *group* (F(2,91.0) = 2.030, *p* = 0.14), and the interaction effect between *item set* *group* and *construct* (F(10,5521.8) = 0.81, *p* = 0.62) were not significant, indicating that there were no differences in the item ratings between the three item set groups.

To evaluate whether the item set groups were also comparable with respect to potentially moderating variables, chi squared tests were conducted comparing the groups with respect to their *pain status* and *gender.* A one-way ANOVA was conducted to compare the *age* of the three item set groups. For the analyses on pain status we created two *pain* groups. Pain group 1 consisted of participants with a pain grade of 0, 1 or 2 (*n* = 70). Pain Group 2 consisted of participants with a pain grade of 3 or 4 (*n* = 24)). The three item set groups did not differ with respect to pain (*χ*^2^(2) = 0.22, *p* = 0.89), gender ( *χ*^2^(2) = 1.62, *p* = 0.44), or age (*F*(2,91) = 0.62, *p* = 0.54).

1. **Influence of moderators**

We checked whether the factors *age*, *gender* and *pain group* could moderate the relationship between the *constructs* (pain catastrophizing, pain-related worrying, pain-related disability, pain-related distress, pain vigilance, pain severity) and the score allocated to the items with linear mixed effect models as implemented in the R package lmerTest (version 3.1.0; Kuznetsova, Brockhoff, & Christensen, 2017). Satterthwaite’s approximation was used to obtain the degrees of freedom (SAS Technical Report R-101, 1978). Model assumptions of  independence, normality and homogeneity of variance were checked. Significance was evaluated at the 5% significance level.

For each catastrophizing measure a model was fitted with *construct*, *age*, *gender, pain group* and the two-way interactions between *construct* and each of the other variables as fixed effects and *subject* and *item* as random effects. Models were initially fitted with maximum likelihood. The fixed effects structure was simplified using stepwise regression (backward elimination) based on likelihood ratio tests. Once the final model was selected, the model was refitted with restricted maximum likelihood (REML).

1. **AEQ**

Table 1. Model selection. The full model contains the main effect of Construct, Gender, Pain group and age and the two-way interactions between construct and the other variables.

|  | **AIC** | **F** | ***p*** |
| --- | --- | --- | --- |
| **Full model** | 6206.94 |  |  |
| **- Construct*Pain group** | 6207.210 | 2.07 | 0.07 |
| **- Pain group** | 6206.89 | 0.26 | 0.61 |
| **- Construct*Age** | 6205.48 | 1.95 | 0.08 |
| **- Age** | 6205.16 | 0.24 | 0.63 |

Table 2. Parameter estimates for the final model. The final model contains the main effects of construct and gender and their interaction effect.

|  | $\hat{\boldsymbol{\beta}}$ | **95% CI** | ***p*** |
| --- | --- | --- | --- |
| **Intercept** | 4.78 | [2.96 to 6.60] | <0.001 |
| **Construct** |  |  |  |
| **Disability vs. Catastrophizing** | -11.10 | [-13.26 to -8.94] | <0.001 |
| **Distress vs. Catastrophizing** | -0.36 | [-2.52 to 1.80] | 0.74 |
| **Severity vs. Catastrophizing** | -10.69 | [-12.85 to -8.53] | <0.001 |
| **Vigilance vs. Catastrophizing** | -8.43 | [-10.59 to -6.27] | <0.001 |
| **Worry vs. Catastrophizing** | 1.66 | [-0.50 to 3.82] | 0.13 |
| **Gender (male vs. female)** | -0.84 | [-3.16 to 1.48] | 0.48 |
| **Construct (disability vs. catastrophizing) * Gender (male vs. female)** | 1.12 | [-1.64 to 3.88] | 0.43 |
| **Construct (distress vs. catastrophizing) * Gender (male vs. female)** | -0.36 | [-3.12 to 2.40] | 0.80 |
| **Construct (severity vs. catastrophizing) * Gender (male vs. female)** | 3.36 | [0.59 to 6.12] | 0.02 |
| **Construct (vigilance vs. catastrophizing) * Gender (male vs. female)** | 2.91 | [0.14 to 5.67] | 0.04 |
| **Construct (worry vs. catastrophizing) * Gender (male vs. female)** | 0.04 | [-2.72 to 2.80] | 0.98 |

Figure 1. Interaction plot illustrating the interaction effect between construct and gender for the AEQ. Point estimates together with their 95% confidence intervals are shown.


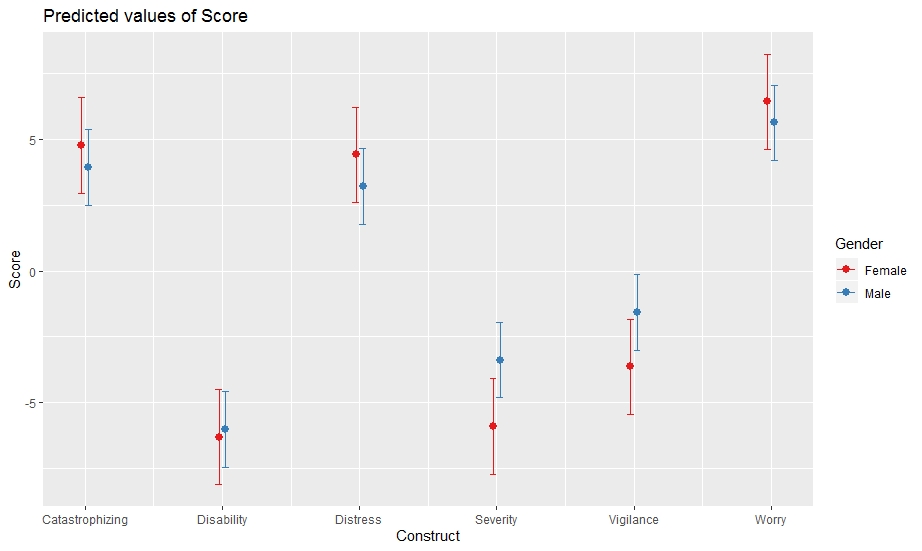


1. **CCSI**

Table 1. Backward model selection. The full model contains the main effect of Construct, Gender, Pain group and age and the two-way interactions between construct and the other variables. At each stage one of the variables was dropped. Models were compared with likelihood ratio tests. If a variable did not significantly improve the fit of the model, it was left out of the final model.

|  | **AIC** | **F** | ***p*** |
| --- | --- | --- | --- |
| **Full model** | 12653.12 |  |  |
| **- Construct*Pain group** | 12646.10 | 0.60 | 0.70 |
| **- Construct*Gender** | 12639.24 | 0.63 | 0.68 |
| **- Pain group** | 12637.65 | 0.42 | 0.52 |
| **- Gender** | 12637.11 | 1.46 | 0.23 |
| **- Construct*Age** | 12634.59 | 1.50 | 0.19 |
| **- Age** | 12633.30 | 0.71 | 0.40 |

Table 2. Parameter estimates for the final model. The final model contains the main effects of construct.

|  | $\hat{\boldsymbol{\beta}}$ | **95% CI** | ***p*** |
| --- | --- | --- | --- |
| **Intercept** | 3.02 | [1.77 to 4.26] | <0.001 |
| **Construct** |  |  |  |
| **Disability vs. Catastrophizing** | -8.28 | [-9.31 to -7.24] | <0.001 |
| **Distress vs. Catastrophizing** | 0.82 | [-0.22 to 1.85] | 0.12 |
| **Severity vs. Catastrophizing** | -4.33 | [-5.37 to -3.30] | <0.001 |
| **Vigilance vs. Catastrophizing** | -5.03 | [-6.07 to -4.00] | <0.001 |
| **Worry vs. Catastrophizing** | 1.11 | [0.08 to 2.15] | 0.03 |

1. **PCS**

Table 1. . Backward model selection. The full model contains the main effect of Construct, Gender, Pain group and age and the two-way interactions between construct and the other variables. At each stage one of the variables was dropped. Models were compared with likelihood ratio tests. If a variable did not significantly improve the fit of the model, it was left out of the final model.

|  | **AIC** | **F** | ***p*** |
| --- | --- | --- | --- |
| **Full model** | 19043.73 |  |  |
| **- Construct*Age** | 19040.98 | 1.45 | 0.20 |
| **- Age** | 19039.06 | 0.08 | 0.78 |
| **- Construct*Pain group** | 19038.22 | 1.84 | 0.10 |
| **- GCPS_Pain** | 19036.43 | 0.21 | 0.65 |
| **- Construct*Gender** | 19037.24 | 2.17 | 0.06 |
| **- Gender** | 19038.11 | 2.91 | 0.09 |

Table 2. Parameter estimates for the final model. The final model contains the main effects of construct.

|  | $\hat{\boldsymbol{\beta}}$ | **95% CI** | ***p*** |
| --- | --- | --- | --- |
| **Intercept** | 1.60 | [0.60 to 2.60] | 0.003 |
| **Construct** |  |  |  |
| **Disability vs. Catastrophizing** | -5.13 | [-5.98 to -4.27] | <0.001 |
| **Distress vs. Catastrophizing** | 4.74 | [3.89 to 5.59] | <0.001 |
| **Severity vs. Catastrophizing** | -0.82 | [-1.67 to 0.04] | 0.06 |
| **Vigilance vs. Catastrophizing** | -3.78 | [-4.63 to -2.92] | <0.001 |
| **Worry vs. Catastrophizing** | 3.90 | [3.05 to 4.76] | <0.001 |

1. **CSQ**

Table 1. . Backward model selection. The full model contains the main effect of Construct, Gender, Pain group and age and the two-way interactions between construct and the other variables. At each stage one of the variables was dropped. Models were compared with likelihood ratio tests. If a variable did not significantly improve the fit of the model, it was left out of the final model.

|  | **AIC** | **F** | ***p*** |
| --- | --- | --- | --- |
| **Full model** |  |  |  |
| **- Construct*Pain group** | 10195.56 | 1.66 | 0.14 |
| **- Pain group** | 10193.82 | 0.13 | 0.72 |

Table 2. Parameter estimates for the final model. The final model contains the main effects of construct, gender and age and the two-way interactions between construct and gender and construct and age.

|  | $\hat{\boldsymbol{\beta}}$ | **95% CI** | ***p*** |
| --- | --- | --- | --- |
| **Intercept** | 3.78 | [0.66 to 6.90] | 0.02 |
| **Construct** |  |  |  |
| **Disability vs. Catastrophizing** | -4.20 | [-7.87 to -0.52] | 0.03 |
| **Distress vs. Catastrophizing** | 3.72 | [0.04 to 7.39] | 0.05 |
| **Severity vs. Catastrophizing** | -2.48 | [-6.15 to 1.20] | 0.19 |
| **Vigilance vs. Catastrophizing** | -9.77 | [-13.45 to -6.10] | <0.001 |
| **Worry vs. Catastrophizing** | -1.53 | [-5.20 to 2.15] | 0.42 |
| **Gender (male vs. female)** | 2.44 | [0.53 to 4.36] | 0.01 |
| **Age** | -0.02 | [-0.10 to 0.05] | 0.58 |
| **Construct (disability vs. catastrophizing) * Gender (male vs. female)** | -0.33 | [-2.62 to 1.95] | 0.77 |
| **Construct (distress vs. catastrophizing) * Gender (male vs. female)** | -3.11 | [-5.40 to -0.83] | 0.008 |
| **Construct (severity vs. catastrophizing) * Gender (male vs. female)** | -0.68 | [-2.97 to 1.60] | 0.56 |
| **Construct (vigilance vs. catastrophizing) * Gender (male vs. female)** | -1.08 | [-3.37 to 1.20] | 0.35 |
| **Construct (worry vs. catastrophizing) * Gender (male vs. female)** | -3.77 | [-6.05 to -1.48] | 0.001 |
| **Construct (disability vs. catastrophizing) * Age** | -0.05 | [-0.14 to 0.04] | 0.26 |
| **Construct (distress vs. catastrophizing) * Age** | 0.03 | [-0.06 to 0.12] | 0.48 |
| **Construct (severity vs. catastrophizing) * Age** | 0.02 | [-0.07 to 0.11] | 0.71 |
| **Construct (vigilance vs. catastrophizing) * Age** | 0.06 | [-0.03 to 0.15] | 0.18 |
| **Construct (worry vs. catastrophizing) * Age** | 0.10 | [0.009 to 0.19] | 0.03 |

Figure 2. Interaction plot illustrating the interaction effect between Gender and Construct for the items of the CSQ. Point estimates together with their 95% confidence intervals are shown.


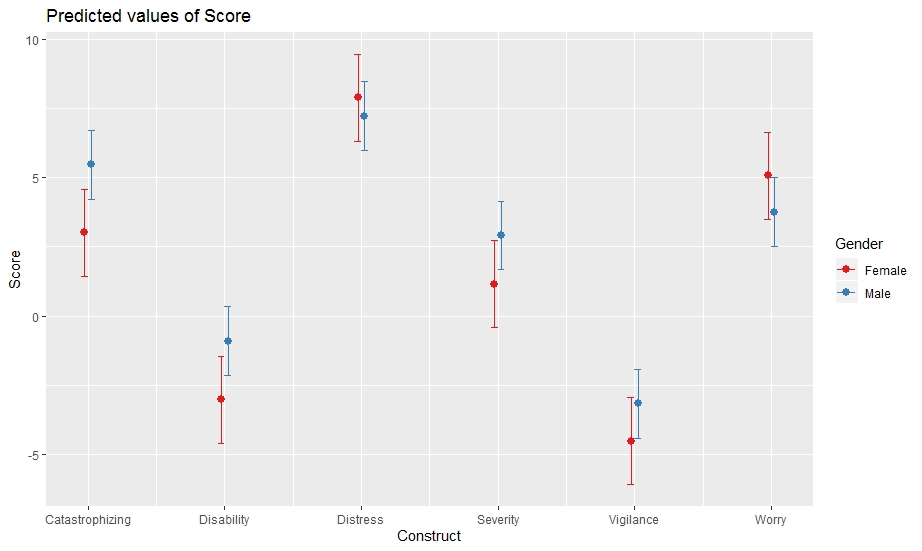


Figure 3. Interaction plot illustrating the interaction effect between Age and Construct for the CSQ. Point estimates together with their 95% confidence intervals are shown.


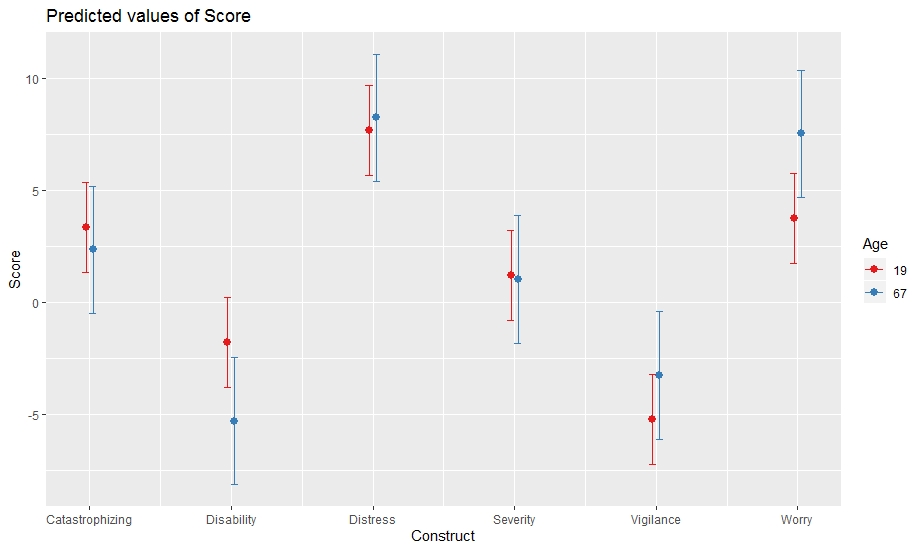


1. **PCL**

Table 1. . Backward model selection. The full model contains the main effect of Construct, Gender, Pain group and age and the two-way interactions between construct and the other variables. At each stage one of the variables was dropped. Models were compared with likelihood ratio tests. If a variable did not significantly improve the fit of the model, it was left out of the final model.

|  | **AIC** | **F** | ***p*** |
| --- | --- | --- | --- |
| **Full model** | 22861.11 |  |  |
| **- Construct*Age** | 22857.60 | 1.30 | 0.26 |
| **- Age** | 22856.73 | 1.14 | 0.29 |

Table 2. Parameter estimates for the final model. The final model contains the main effects of construct, gender and pain group and the two-way interactions between construct and gender and construct and pain group.

|  | $\hat{\boldsymbol{\beta}}$ | **95% CI** | ***p*** |
| --- | --- | --- | --- |
| **Intercept** | -4.82 | [-6.47 to -3.17] | <0.001 |
| **Construct** |  |  |  |
| **Disability vs. Catastrophizing** | 0.72 | [-0.80 to 2.24] | 0.35 |
| **Distress vs. Catastrophizing** | 5.22 | [3.71 to 6.74] | <0.001 |
| **Severity vs. Catastrophizing** | 0.96 | [-0.56 to 2.48] | 0.22 |
| **Vigilance vs. Catastrophizing** | 0.66 | [-0.85 to 2.18] | 0.39 |
| **Worry vs. Catastrophizing** | 4.41 | [2.89 to 5.93] | <0.001 |
| **Gender (male vs. female)** | 2.63 | [0.99 to 4.26] | 0.002 |
| **Pain group (group 1 vs. group 2)** | 1.27 | [-0.54 to 3.08] | 0.17 |
| **Construct (disability vs. catastrophizing) * Gender (male vs. female)** | -2.53 | [-4.25 to -0.80] | 0.004 |
| **Construct (distress vs. catastrophizing) * Gender (male vs. female)** | -2.20 | [-3.93 to -0.47] | 0.01 |
| **Construct (severity vs. catastrophizing) * Gender (male vs. female)** | -1.16 | [-2.89 to 0.57] | 0.19 |
| **Construct (vigilance vs. catastrophizing) * Gender (male vs. female)** | -0.35 | [-2.08 to 1.38] | 0.69 |
| **Construct (worry vs. catastrophizing) * Gender (male vs. female)** | -1.04 | [-2.77 to 0.69] | 0.24 |
| **Construct (disability vs. catastrophizing) * Pain group (group 1 vs. group2)** | -2.67 | [-4.58 to -0.75] | 0.006 |
| **Construct (distress vs. catastrophizing) * Pain group (group 1 vs. group2)** | 0.15 | [-1.77 to 2.06] | 0.88 |
| **Construct (severity vs. catastrophizing) * Pain group (group 1 vs. group2)** | -0.66 | [-2.57 to 1.26] | 0.50 |
| **Construct (vigilance vs. catastrophizing) * Pain group (group 1 vs. group2)** | 0.55 | [-1.36 to 2.47] | 0.57 |
| **Construct (worry vs. catastrophizing) * Pain group (group 1 vs. group2)** | 0.65 | [-1.26 to 2.57] | 0.50 |

Figure 4. Interaction plot of the interaction effect between construct and pain group for the PCL. Point estimates together with their 95% confidence intervals are shown.


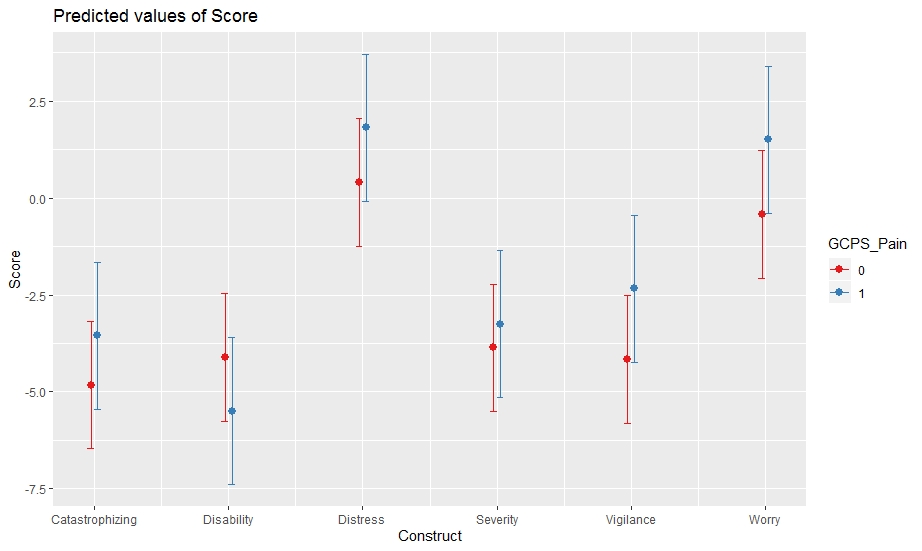


Figure 5. Interaction plot of the interaction effect between gender and construct for the PCL. Point estimates together with their 95% confidence intervals are shown.


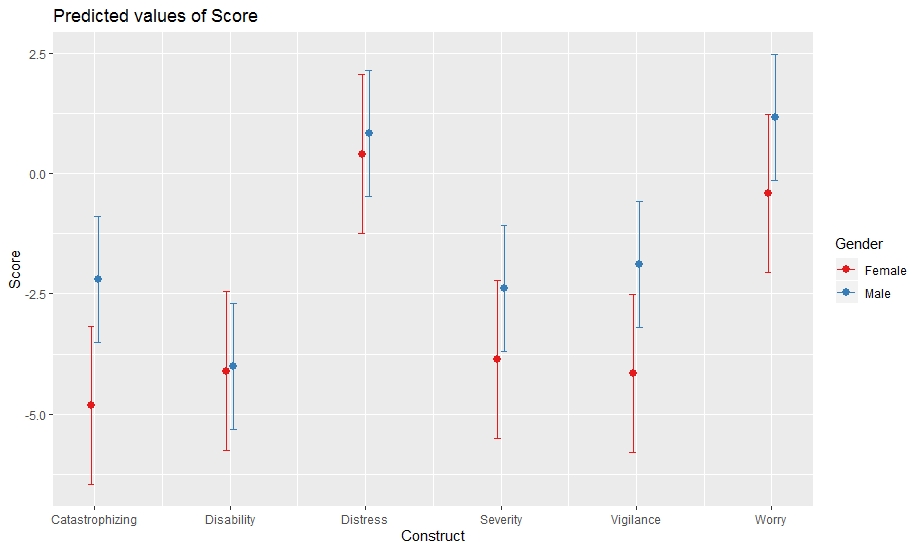


1. **PRSS**

Table 1. . Backward model selection. The full model contains the main effect of Construct, Gender, Pain group and age and the two-way interactions between construct and the other variables. At each stage one of the variables was dropped. Models were compared with likelihood ratio tests. If a variable did not significantly improve the fit of the model, it was left out of the final model.

|  | **AIC** | **F** | ***p*** |
| --- | --- | --- | --- |
| **Full model** | 13956.80 |  |  |
| **- Construct*Gender** | 13949.86 | 0.61 | 0.69 |
| **- Construct*Pain group** | 13944.17 | 0.86 | 0.51 |
| **- Pain group** | 13942.37 | 0.20 | 0.65 |
| **- Gender** | 13943.55 | 3.22 | 0.08 |

Table 2. Parameter estimates for the final model. The final model contains the main effects of construct and age and their interaction effect.

|  | $\hat{\boldsymbol{\beta}}$ | **95% CI** | ***p*** |
| --- | --- | --- | --- |
| **Intercept** | 3.75 | [0.78 to 6.71] | 0.01 |
| **Construct** |  |  |  |
| **Disability vs. Catastrophizing** | -6.51 | [-9.73 to -3.29] | <0.001 |
| **Distress vs. Catastrophizing** | -2.38 | [-5.60 to 0.84] | 0.15 |
| **Severity vs. Catastrophizing** | 0.11 | [-3.11 to 3.33] | 0.95 |
| **Vigilance vs. Catastrophizing** | -8.76 | [-11.98 to -5.54] | <0.001 |
| **Worry vs. Catastrophizing** | -6.28 | [-9.50 to -3.06] | <0.001 |
| **Age** | -0.04 | [-0.12 to 0.03] | 0.23 |
| **Construct (disability vs. catastrophizing) * Age** | 0.05 | [-0.03 to 0.13] | 0.24 |
| **Construct (distress vs. catastrophizing) * Age** | 0.12 | [0.03 to 0.20] | 0.006 |
| **Construct (severity vs. catastrophizing) * Age** | 0.02 | [-0.06 to 0.11] | 0.61 |
| **Construct (vigilance vs. catastrophizing) * Age** | 0.10 | [0.01 to 0.18] | 0.02 |
| **Construct (worry vs. catastrophizing) * Ge Age** | 0.17 | [0.09 to 0.25] | <0.001 |

Figure 6. Interaction plot illustrating the interaction effect between construct and age for the PRSS. Point estimates together with their 95% confidence intervals are shown.


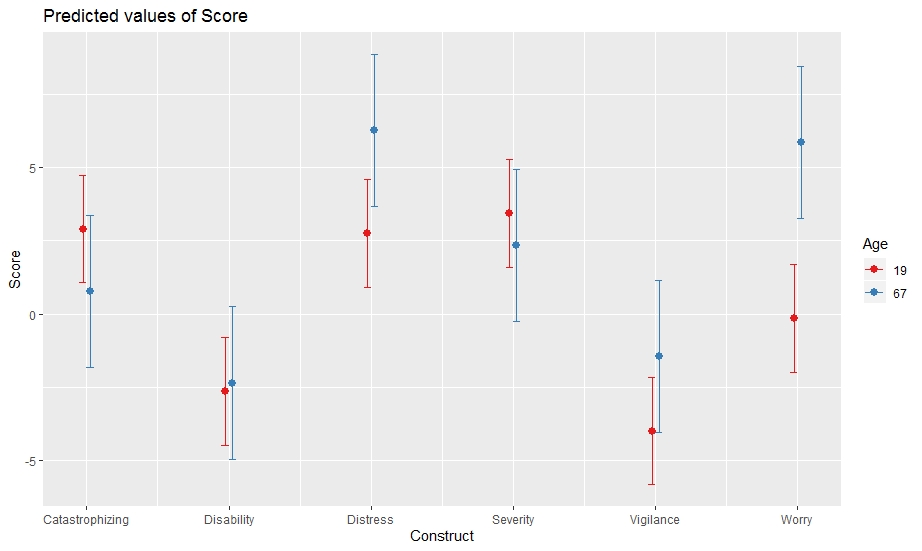

Supplement: File S2 [file peerj-08-8643-s003.docx]
